# Supplementary material for: Analysis of Fowl Adenovirus 4 Transcriptome by De Novo ORF Prediction Based on Corrected Nanopore Full-Length cDNA Sequencing Data
Source: Viruses. 2023 Feb 14;15(2):529. doi: 10.3390/v15020529 (PMC9962806; doi:10.3390/v15020529)
Supplement: Supplementary file 1 [file viruses-15-00529-s001.zip › viruses-2168784-supplementary.pdf]

Table S1. Statistics on the nanopore full-length cDNA sequencing

time point post FAdV-4 infection (hours)	12	18	26	combined
total reads	8,267,472	6,963,460	4,345,719	
full length reads	5,766,328	5,374,181	2,441,748	
ratio of the number of full length reads to that of total reads	69.7%	77.2%	56.2%	
average full length cDNA read length (bp)	1473	1615	1716	
Full length cDNA N10 size (bp)	4249	3672	4264	
Full length cDNA N50 size (bp)	2183	1900	2123	
Full length cDNA N90 size (bp)	921	831	884	
reads after host cDNA removing and nucleotide correction	174,839	417,938	298,892	891,669
reads mapped to FAdV-4 genome	60,661	309,978	252,524	623,158
ratio of the copy number of viral transcripts to that of total ones	1.1%	5.8%	10.3%	
copy number of perl script grouped transcripts	60,517	308,517	251,106	620,142
copy number of the viral transcripts encoding protein larger than 50 aa	58,584	289,460	238,253	586,292
copy number of the transcripts encoding found 534 viral ORFs	57,129	282,539	231,692	571,790
copy number of the transscripts encoding selected 105 viral ORFs	52,304	244,029	191,649	487,722
copy number ot the transcripts encoding selected 81 viral ORFs	51,588	239,883	188,839	480,127

The N10 cDNA size is defined as the length of the smallest cDNA S in the sorted list of all cDNAs where the cumulative length from the largest cDNA to cDNA S is at least 10% of the total length, so are the N50 or N90 sizes.

Table S2. Primers used in RT-PCR for mRNA validation

ORF name	Primer name	Sequence	primer binding sites in genome (nt)	genome product (bp)	cDNA product (bp)
ORF1-s100aa	2208ORF1-s100aaS1	atcgcggtga agtgaaggtg	1064 .. 1083	561	487
	2208ORF1-s100aaS2	tcatcccagg cgttaagcag	1605 .. 1624		
ORF13-e299aa	2208ORF13-e299aaS1	cgtcgggtac atacggagac t	6364 .. 6384	852	766
	2208ORF13-e299aaS2	tggagaagct gtccgcgata	5533 .. 5552		
ORF12-e305aa	2208ORF12-e305aaS1	cccacattgc accactcctt	16,988 .. 17,007	10610	777
	2208ORF12e305aaS2	cgaatgcgag acctacgg	6398 .. 6415		
DBP-h574aa	2208DBP-h574aaS1	ctctgttata ggcgacacct cg	26,978 .. 26,999	2000	747
	2208DBP-h574aaS2	gcgctgaaag tgacgggat	25,000 .. 25,018		
Uexon-h205aa	2208Uexon-h205aaS1	ccggctaggt tcggatagg	30,525 .. 30,543	5252	759
	2208Uexon-h205aaS2	gagacgtgga cggtcgttct a	25,292 .. 25,312		
ORF19A-e865aa	2208ORF19A-e865aaS1	tccatccgaa gactgatcca g	39,984 .. 40,004	597	516
	2208ORF19A-e865aaS2	gatagtgacg gcgtggtaag c	40,560 .. 40,580		
ORF4-h171aa	2208ORF4-h171aaS1	gaacacgaca tgggaggcg	42,843 .. 42,861	601	538
	2208ORF4-h171aaS2	ggattgcggt tggttagtct gt	43,422 .. 43,443		

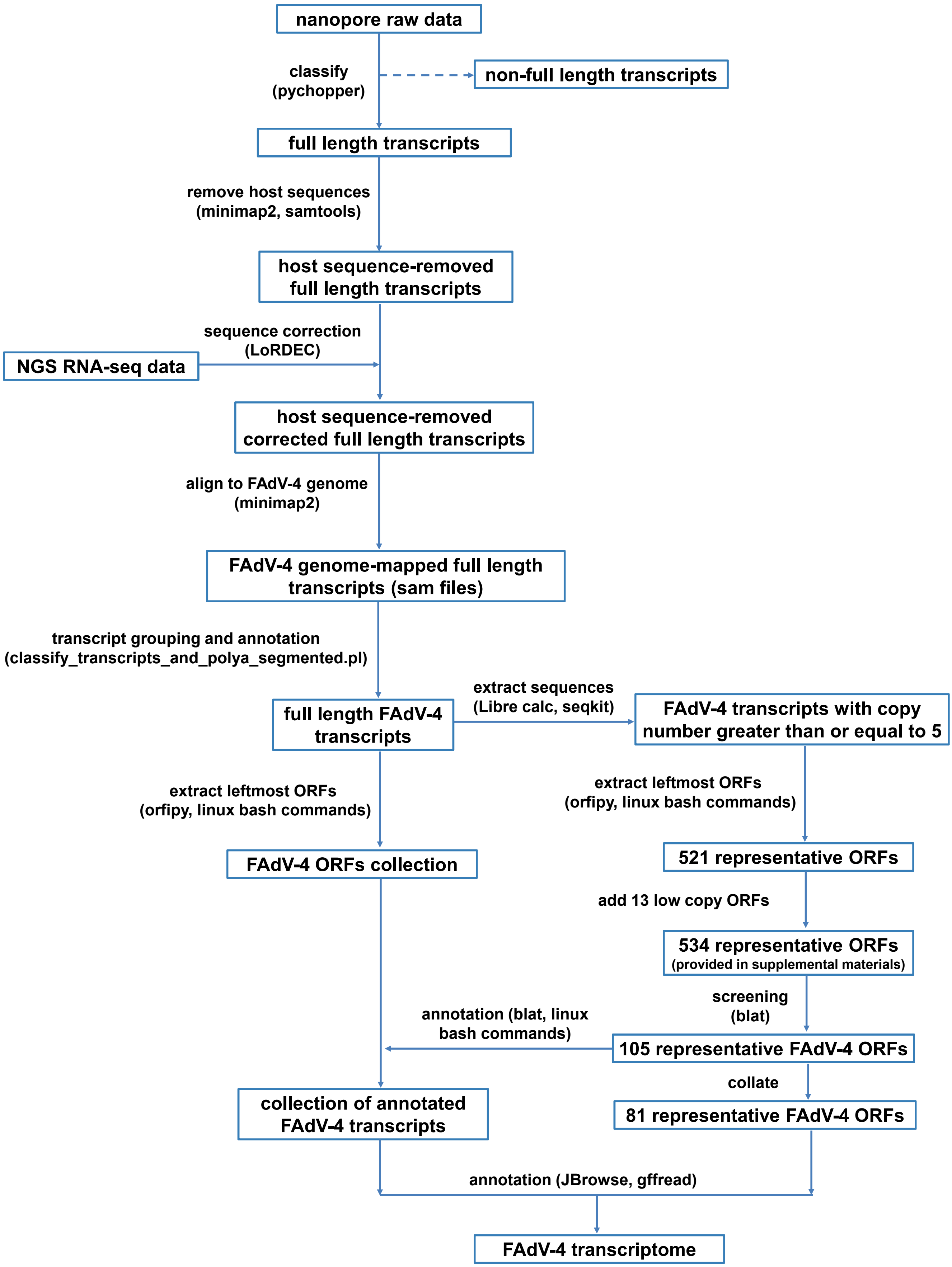

Figure S1. Workflow for FAdV-4 transcriptome analysis

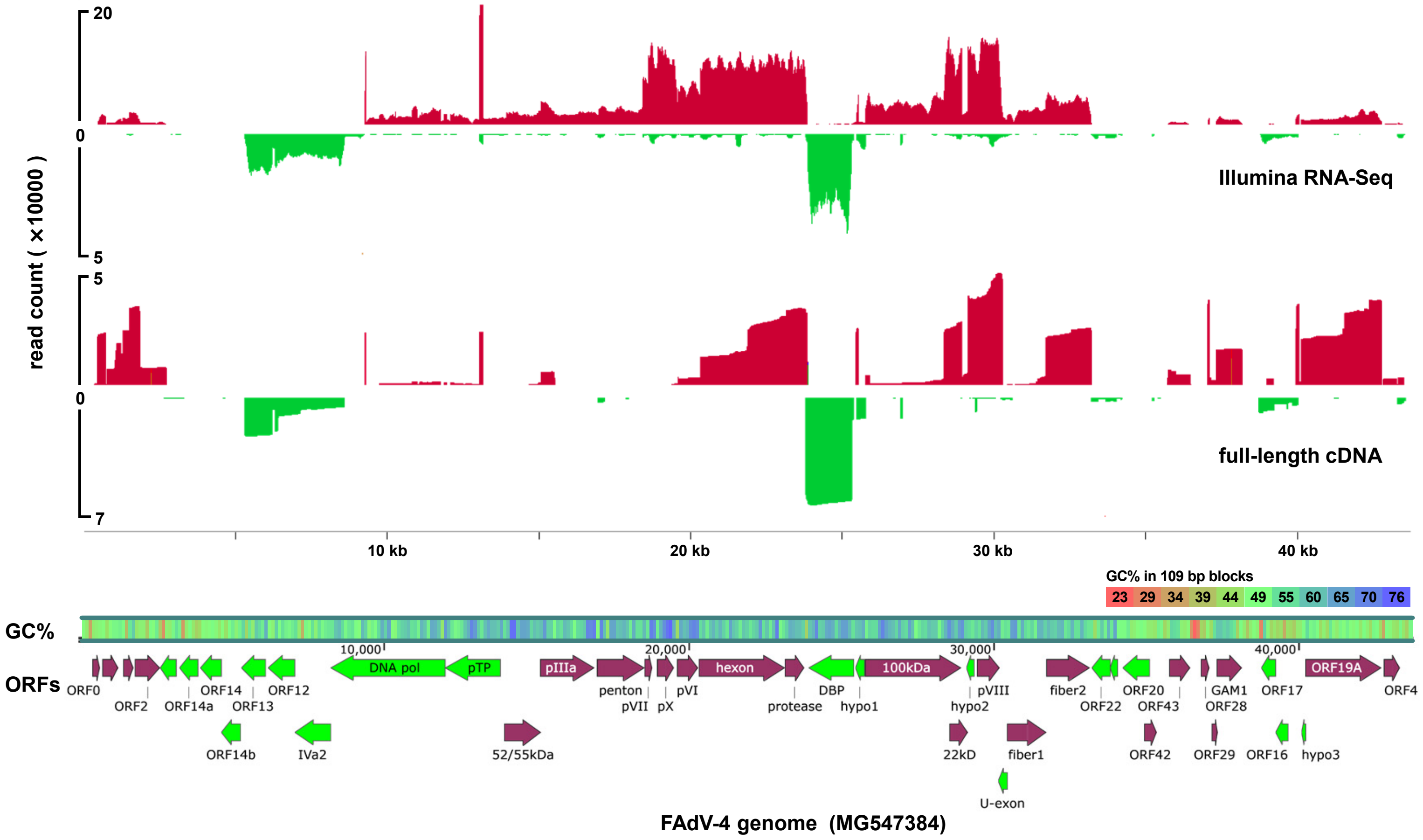

Figure S2. Reads coverage on the genome of fowl adenovirus 4 (FAdV-4). Chicken LMH cells were infected with wt-FAdV-4 at an MOI of 400 viral particles (vp) per cell for 2 hours. RNA was extracted at 12, 18 or 26 hours post infection and subjected to Illumina strand-specific RNA-seq or nanopore full length PCR-cDNA sequencing as described in the Materials and Methods section. The nanopore sequencing results were corrected with Illumina RNA-seq data, both of which were aligned to FAdV-4 genome (GenBank MG547384) and visualized in Integrative Genomics Viewer (IGV). The coverage tracks for the data at 18 hours post infection were shown on the top. The heat map of GC content of the genome was drawn by using pDRAW32 software (www.acaclone.com) and shown in the middle. The original ORFs in the genome was annotated on the bottom.

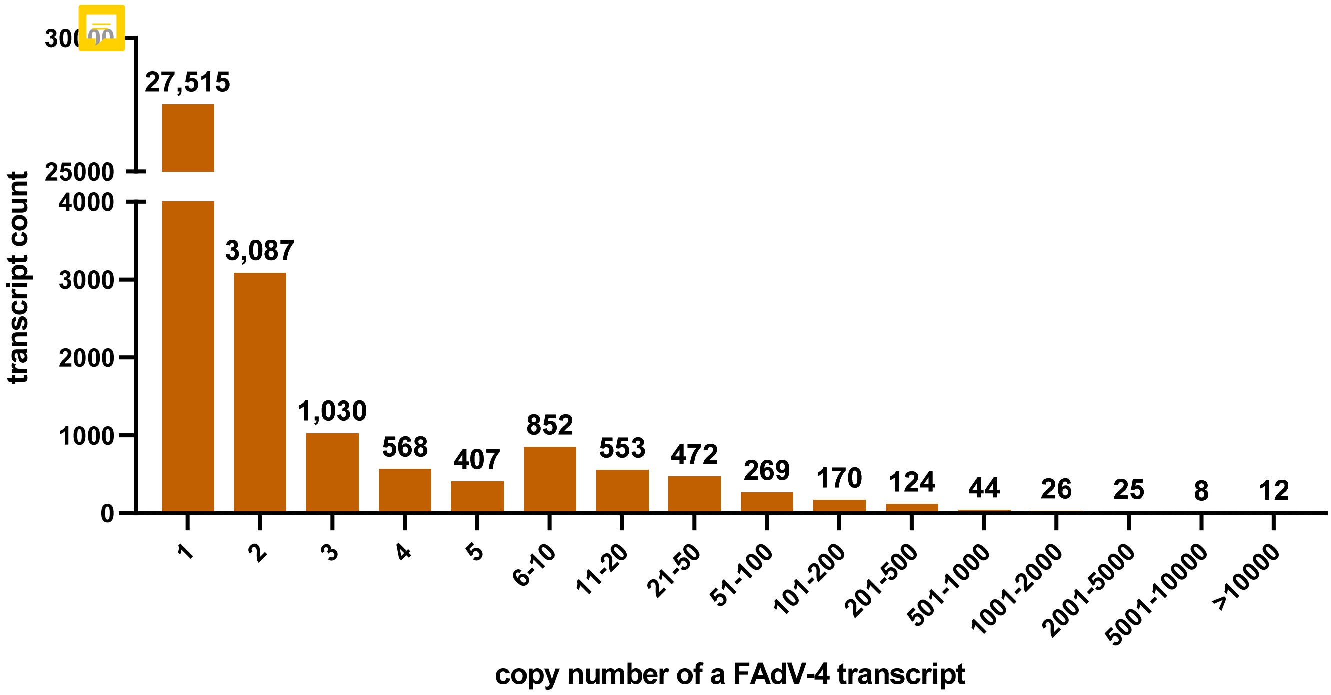

Figure S3. Frequency distribution histogram on the copy numbers of FAdV-4 transcripts (full length cDNAs)

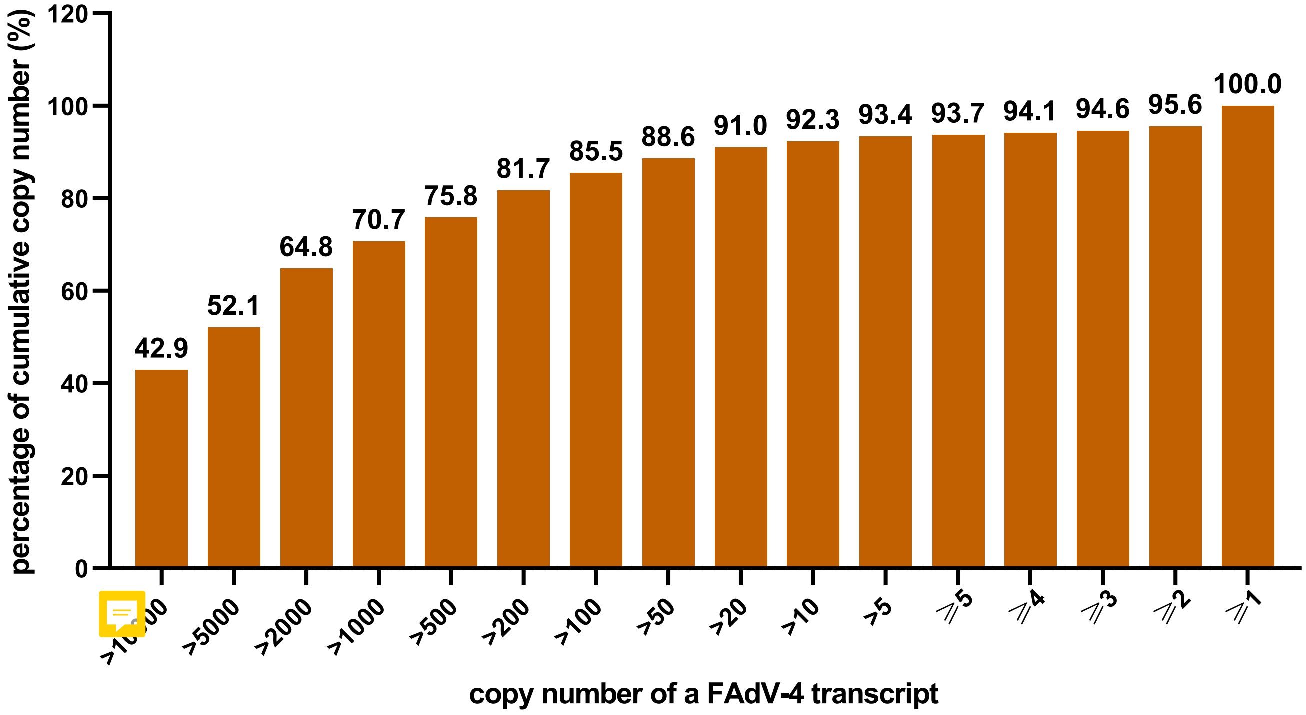

Figure S4. Percentage of cumulative copy number for FAdV-4 transcripts

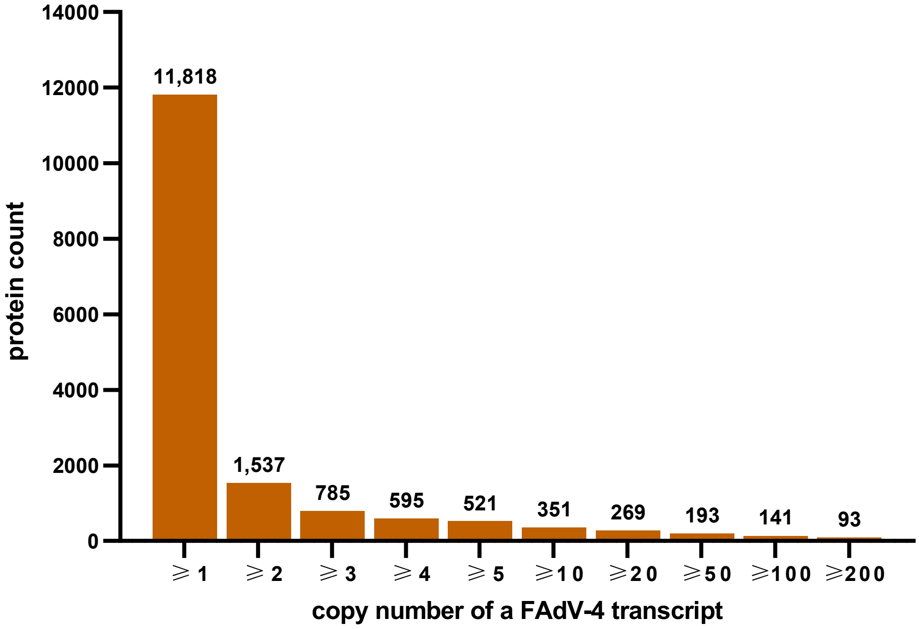

Figure S5. Protein encoding ability of FAdV-4 transcripts

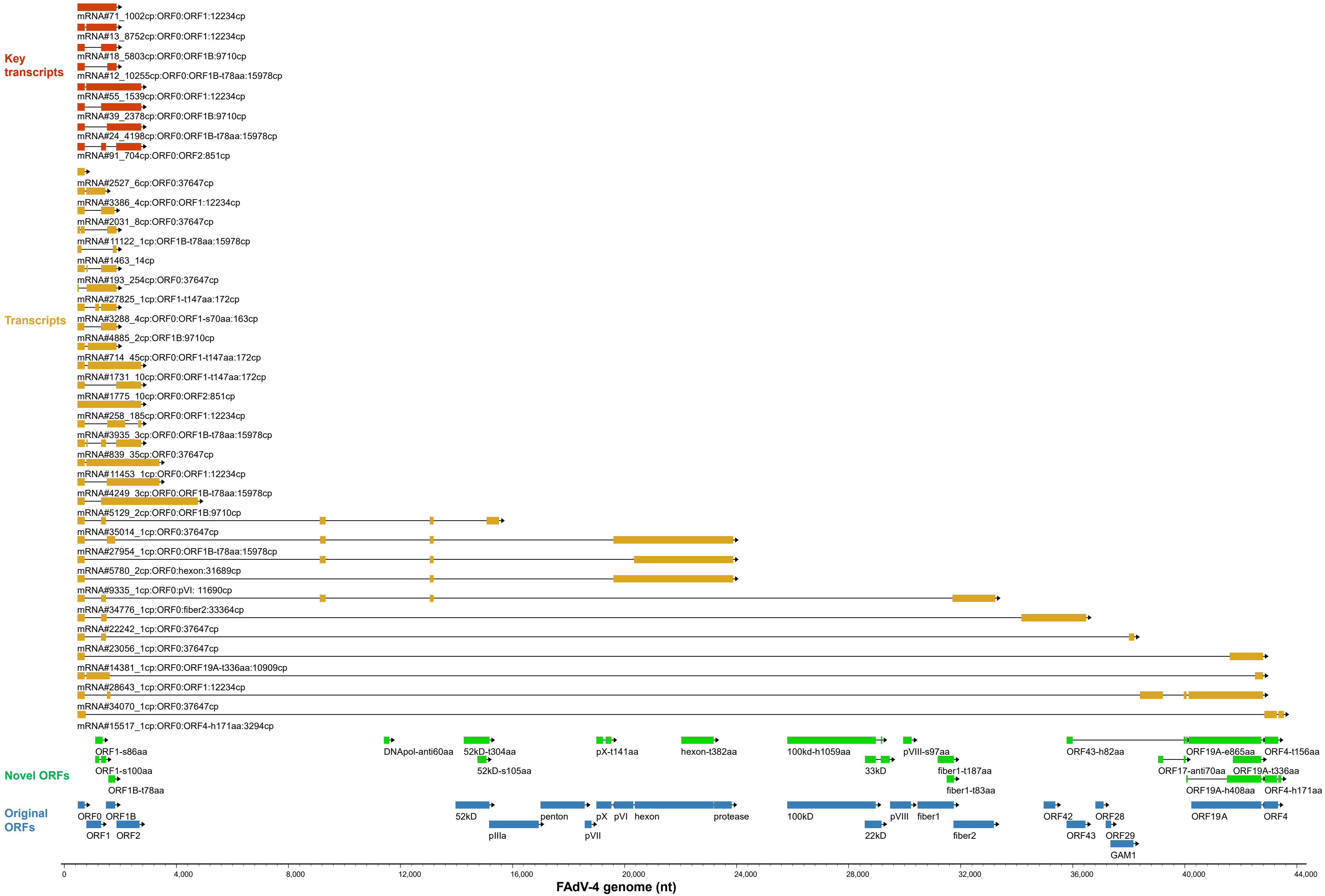

Figure S6. Transcripts that started from the major transcription start site of 466-nt on the forward strand. A virus ORF was encoded by several transcripts, and the transcripts that accounted for a high copy proportion of an ORF were selected as the key transcripts. Shown were also transcripts that met one of the following requirements: the second or the third most abundant transcripts for an ORF, representatives for an ORFs whose key transcript started from another TSS, or representative transcripts for typical splicing sites or other major transcription termination sites (TSS). The sum of the copy numbers of all transcripts that encoded an ORF was calculated and defined as the copy number of the ORF, which was appended to the end of the ORF name. Some transcripts were not annotated with an ORF name since they did not encode any ORF or encoded a protein that was not included in the 105 selected ORFs. Original ORFs: the ORFs that had been annotated in previous publications. Novel ORFs: the ORFs that were newly found in this study. (Transcripts in Figure S7-S16 were similarly annotated)

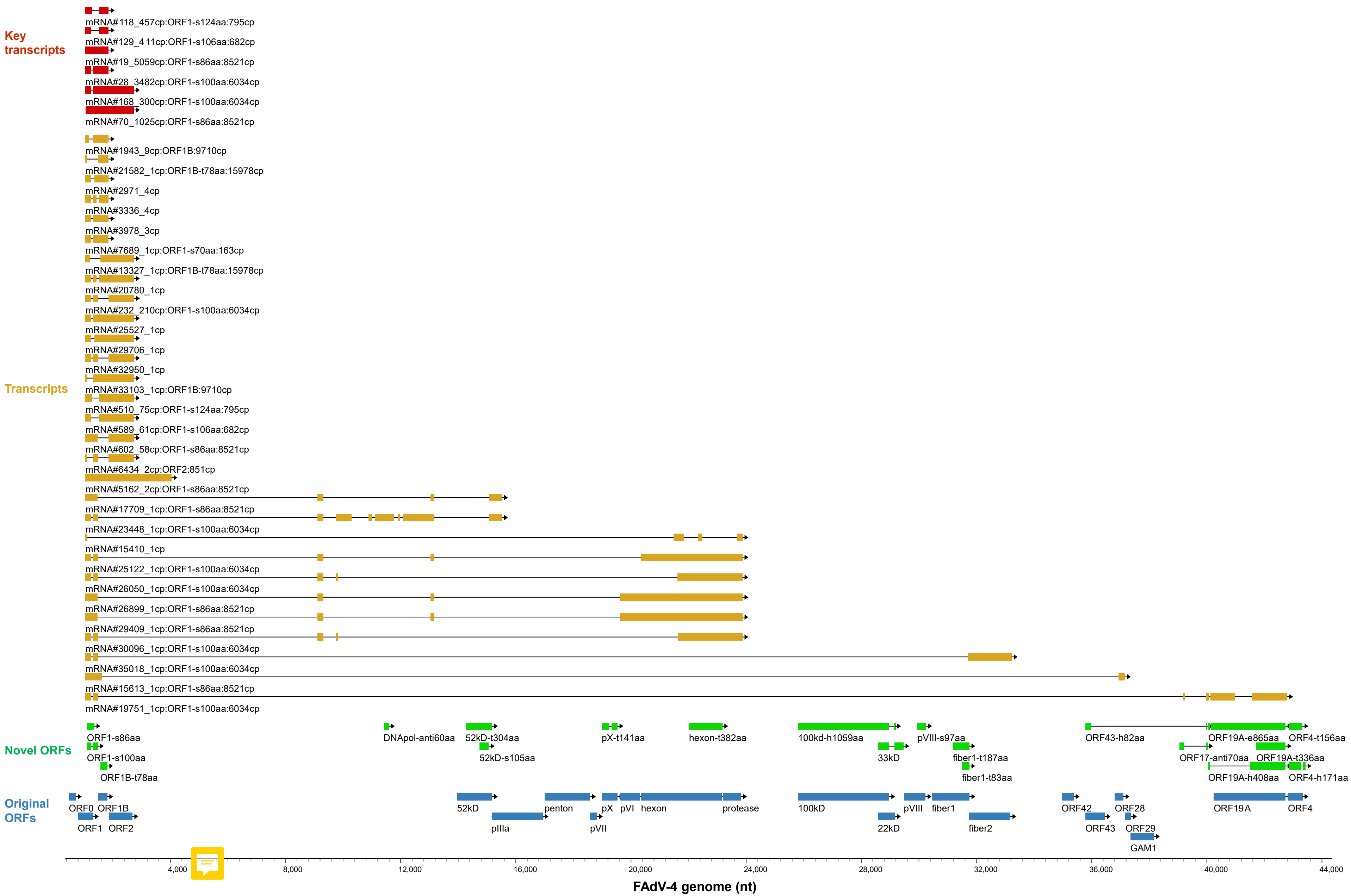

Figure S7. Transcripts that started from the major transcription start site of 1057-nt on the forward strand. The related annotation and explanation could be found in the legend of Figure S6.

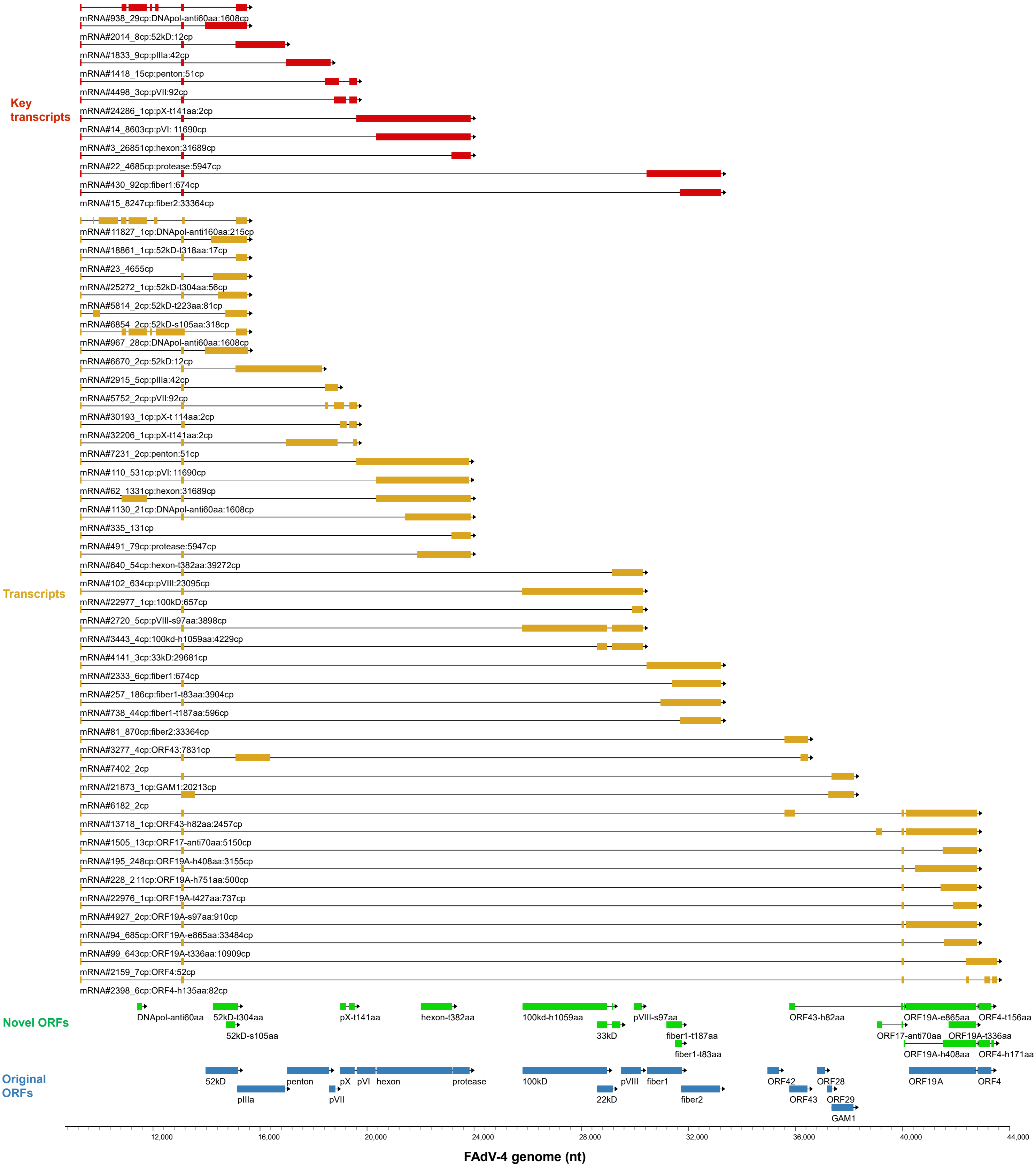

Figure S8. Transcripts that started from the major transcription start site of 9275-nt on the forward strand. The related annotation and explanation could be found in the legend of Figure S6.

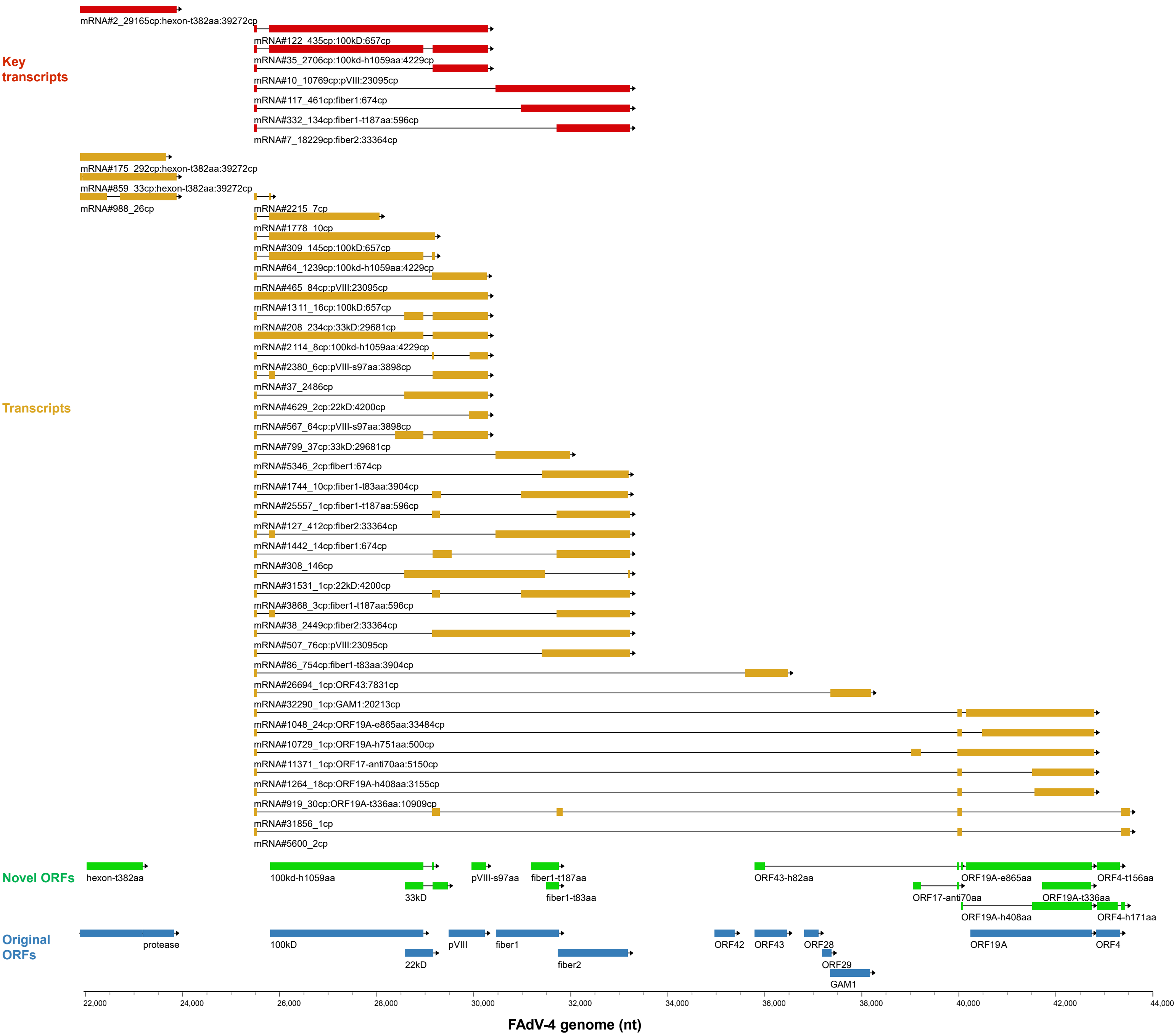

Figure S9. Transcripts that started from the major transcription start site of 21,891-nt and 25,473-nt on the forward strand. The related annotation and explanation could be found in the legend of Figure S6.

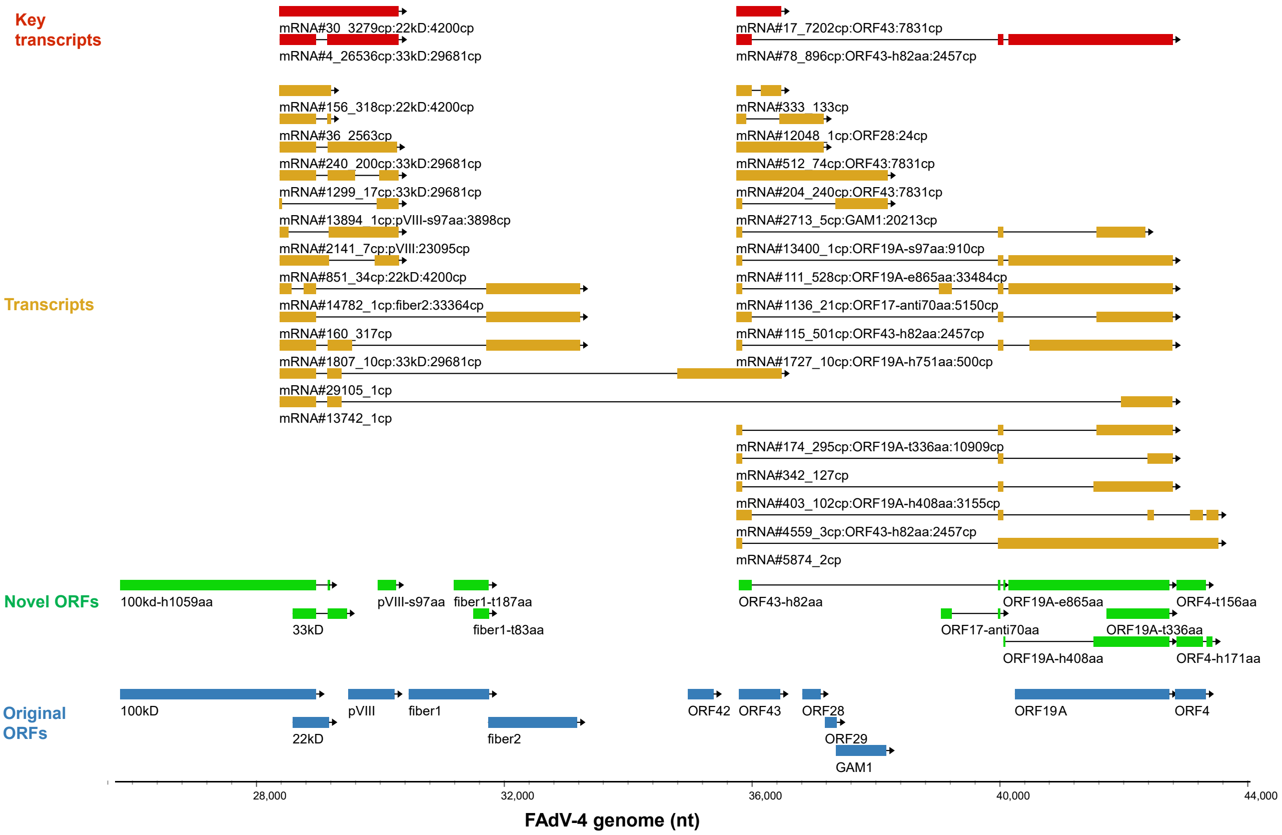

Figure S10. Transcripts that started from the major transcription start site of 28,371-nt and 35,747-nt on the forward strand. The related annotation and explanation could be found in the legend of Figure S6.

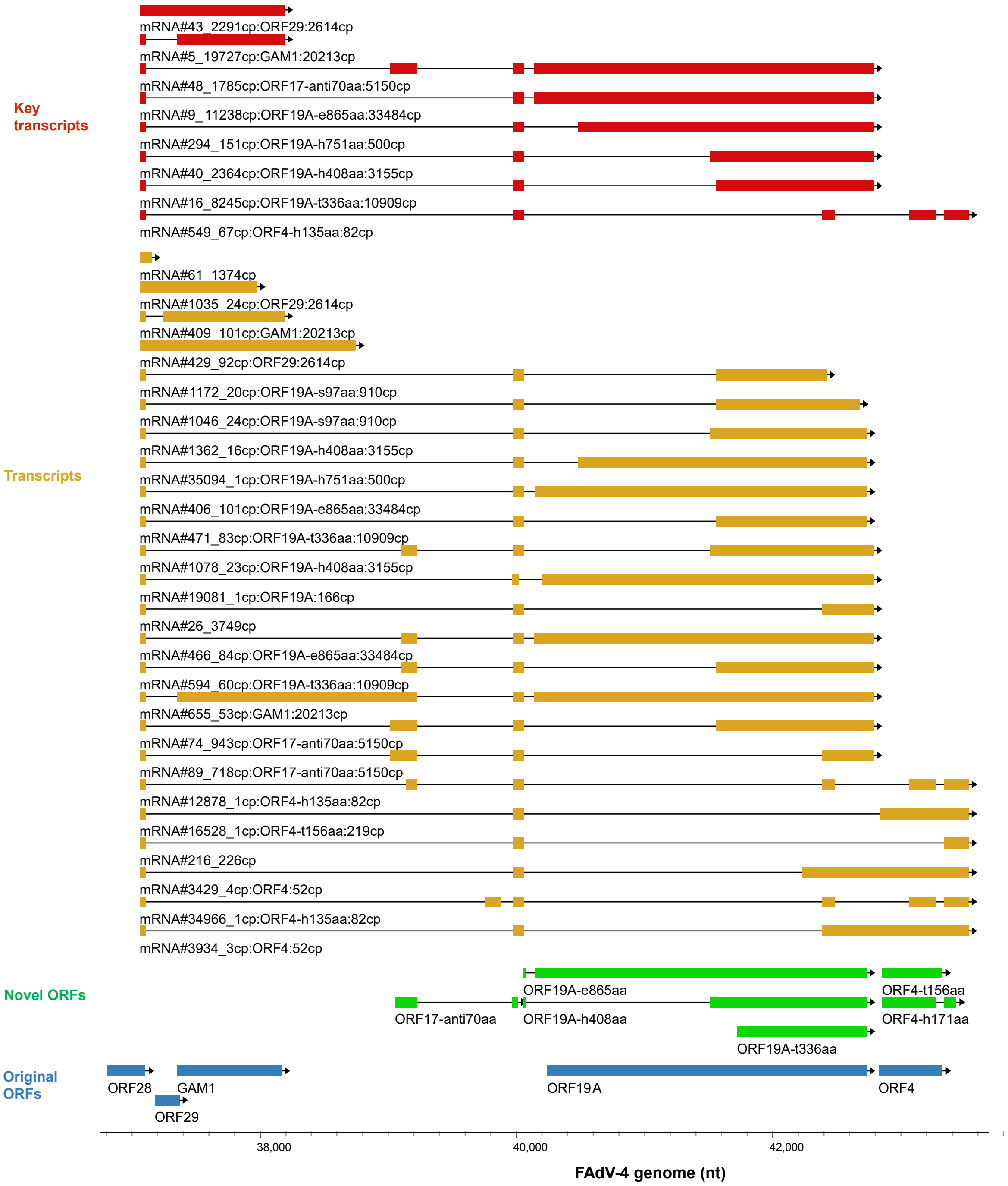

Figure S11. Transcripts that started from the major transcription start site of 37,061-nt on the forward strand. The related annotation and explanation could be found in the legend of Figure S6.

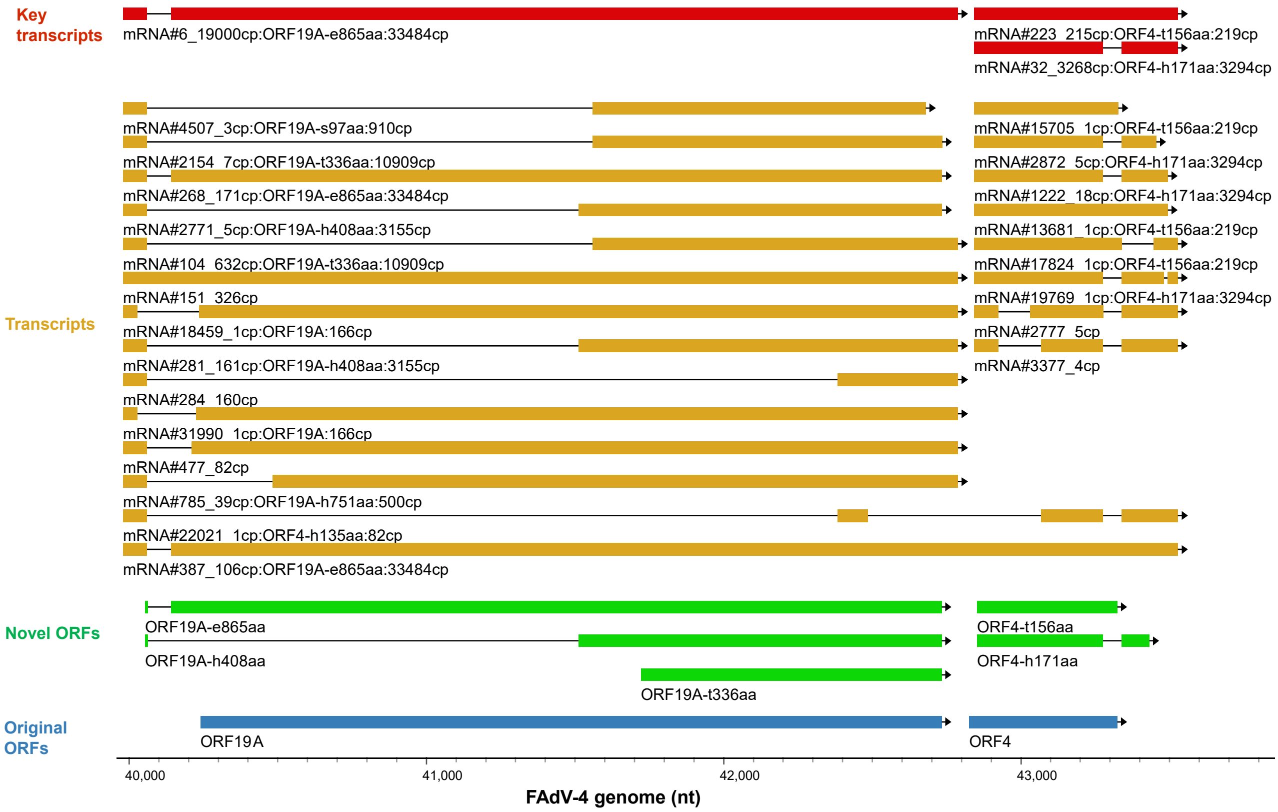

Figure S12. Transcripts that started from the major transcription start site of 39,979-nt and 42,842-nt on the forward strand. The related annotation and explanation could be found in the legend of Figure S6.

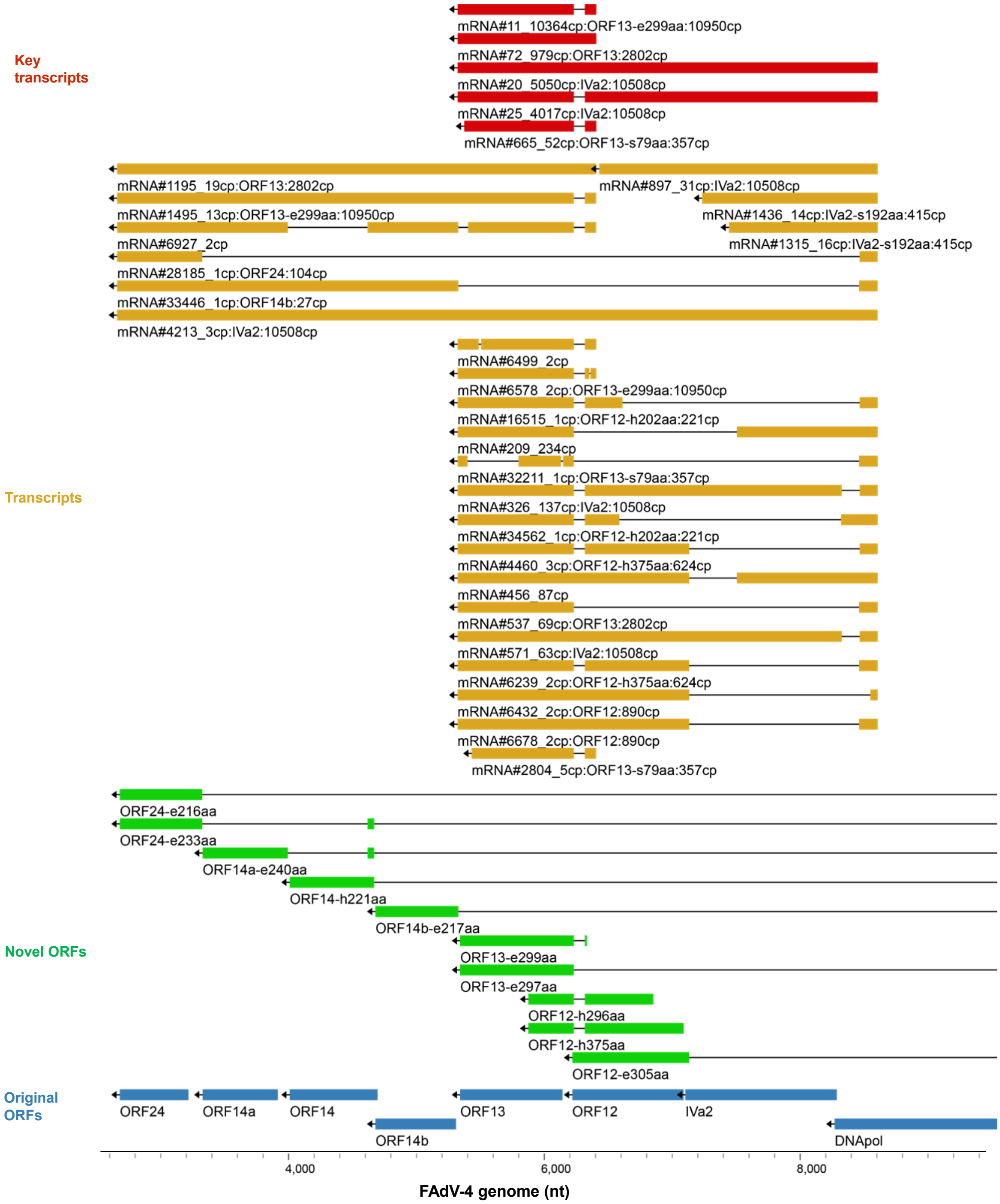

Figure S13. Transcripts that started from the major transcription start site of 6405-nt and 8605-nt on the reverse strand. The related annotation and explanation could be found in the legend of Figure S6.

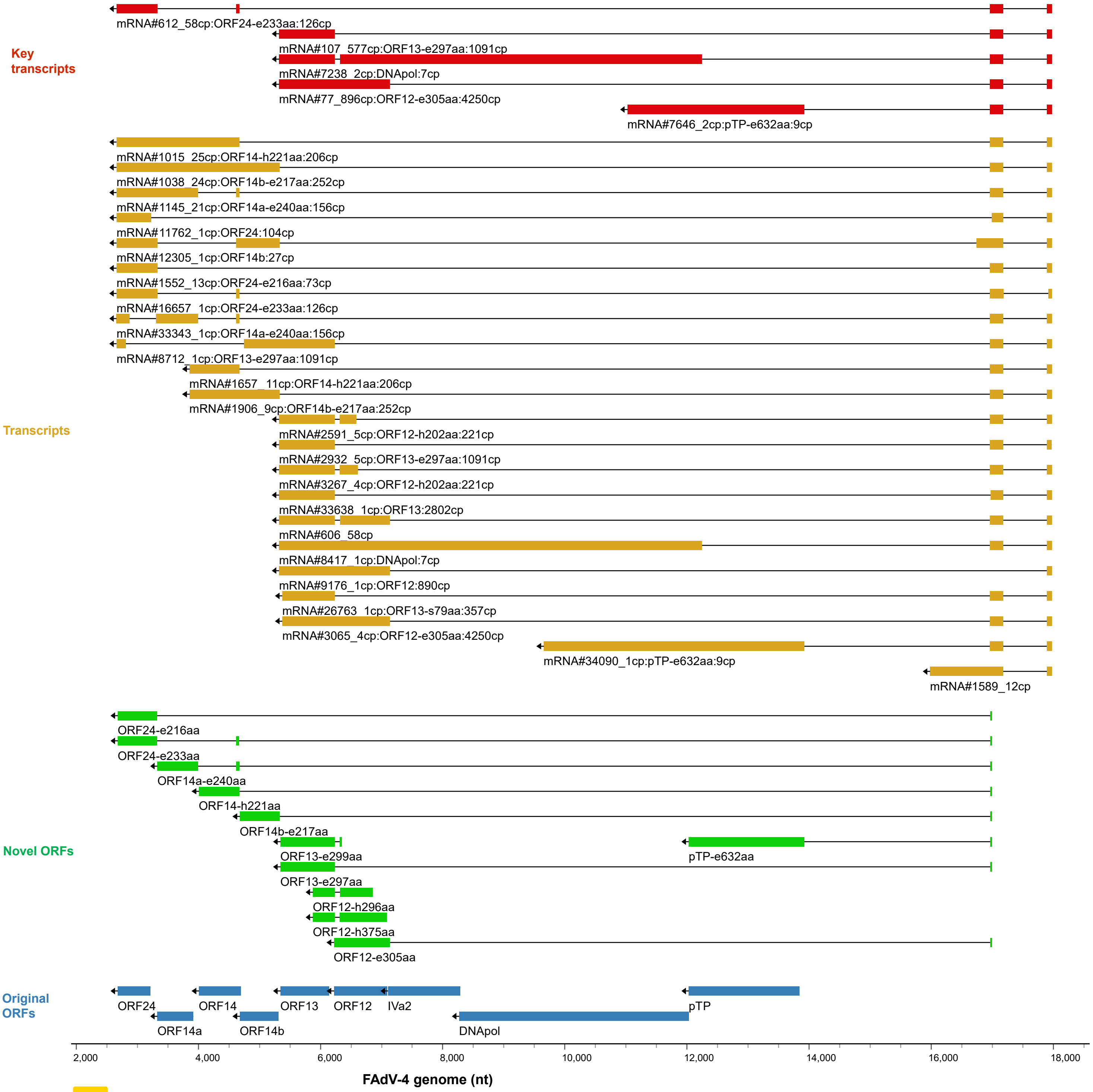

Figure [illegible]4. Transcripts that started from the major transcription start site of 17,972-nt on the reverse strand. The related annotation and explanation could be found in the legend of Figure S6.

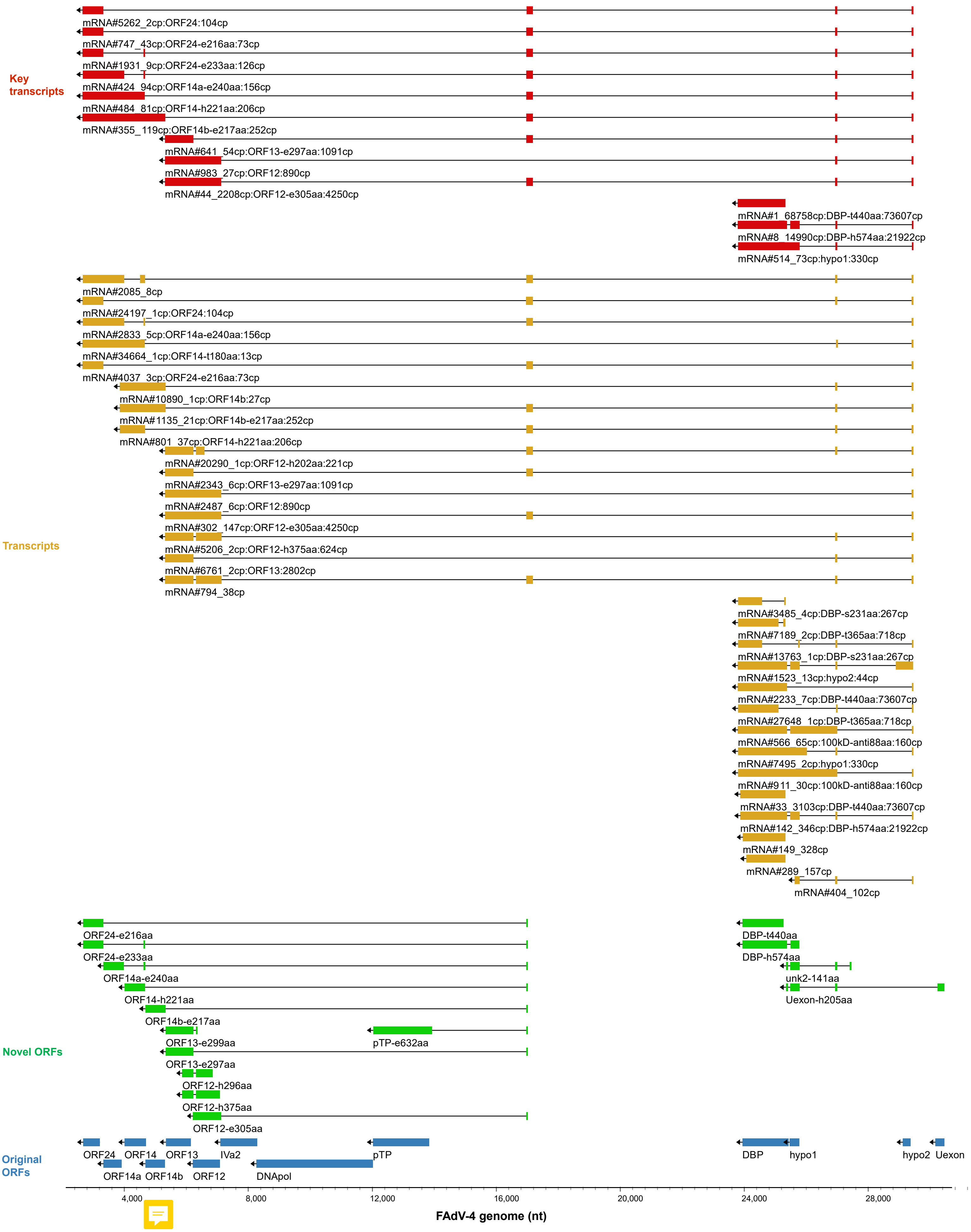

Figure S15. Transcripts that started from the major transcription start site of 25,327-nt and 29,442-nt on the reverse strand. The related annotation and explanation could be found in the legend of Figure S6.

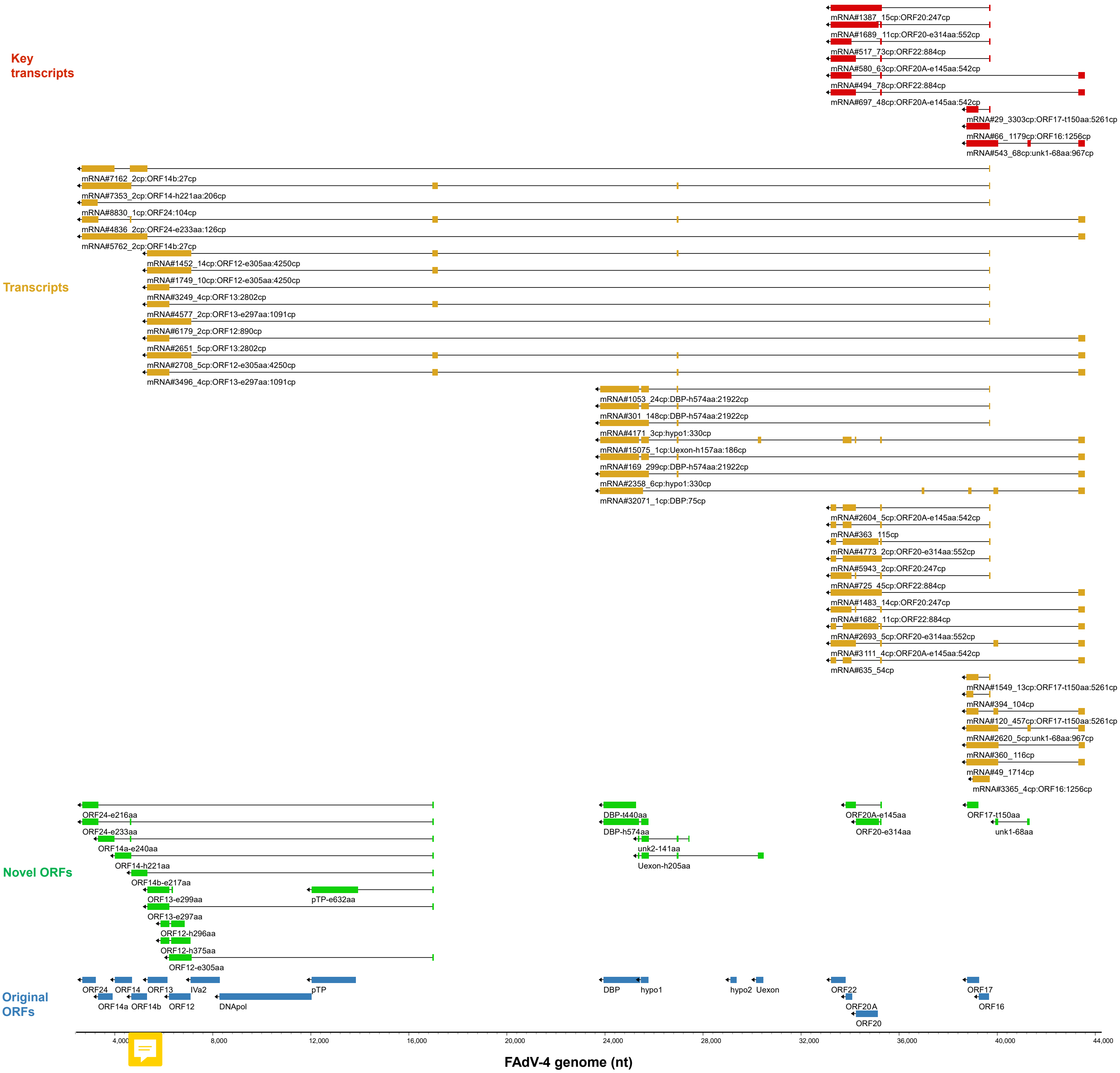

Figure S16. Transcripts that started from the major transcription start site of 39,698-nt and 43,578-nt on the reverse strand. The related annotation and explanation could be found in the legend of Figure S6.

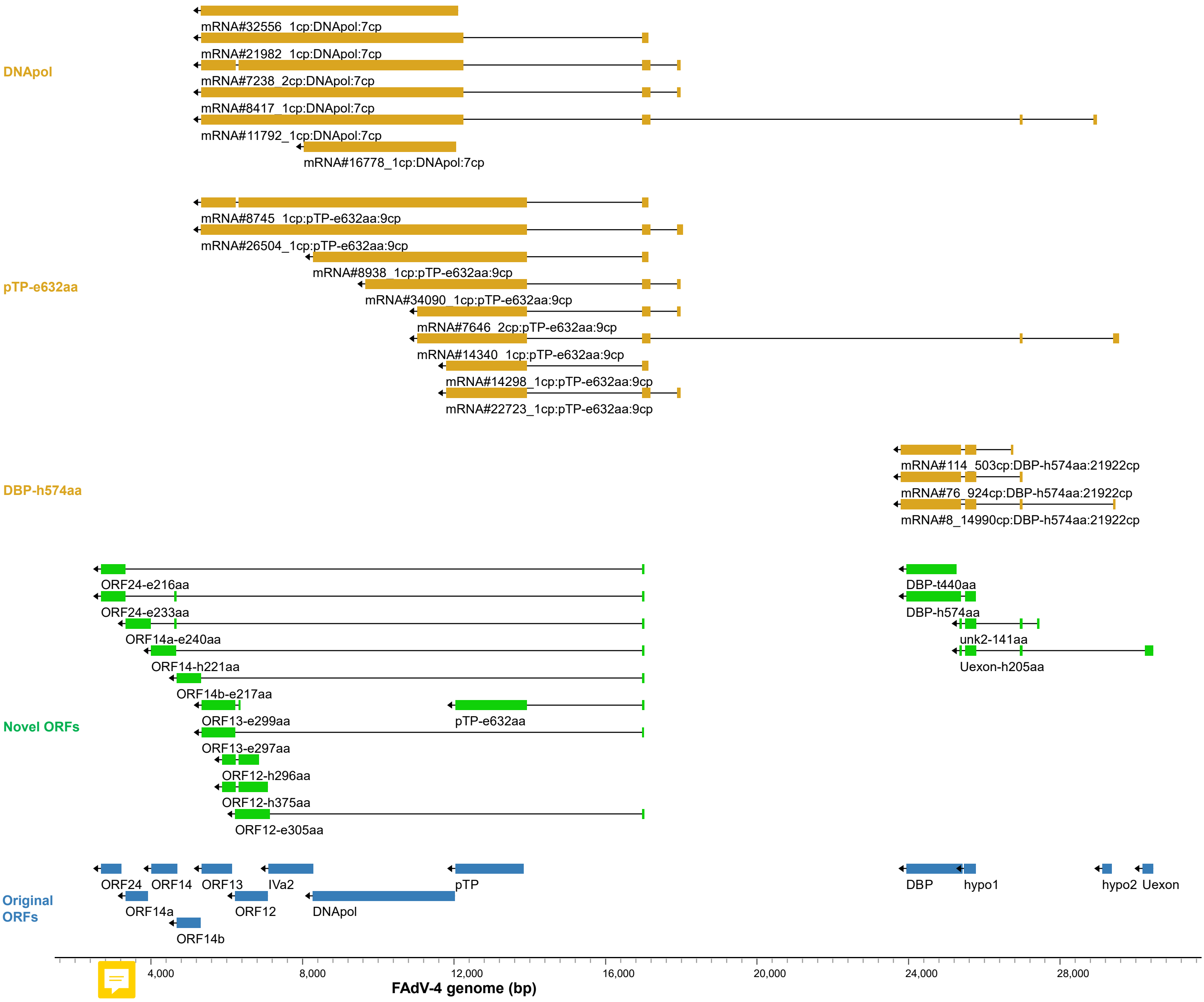

Figure S17. All detected transcripts encoding pTP-e632aa and DNApol. The top 3 transcripts for DBP-h574aa in abundance were displayed to show that different promoters drove the transcription of these virus genome replication related genes.

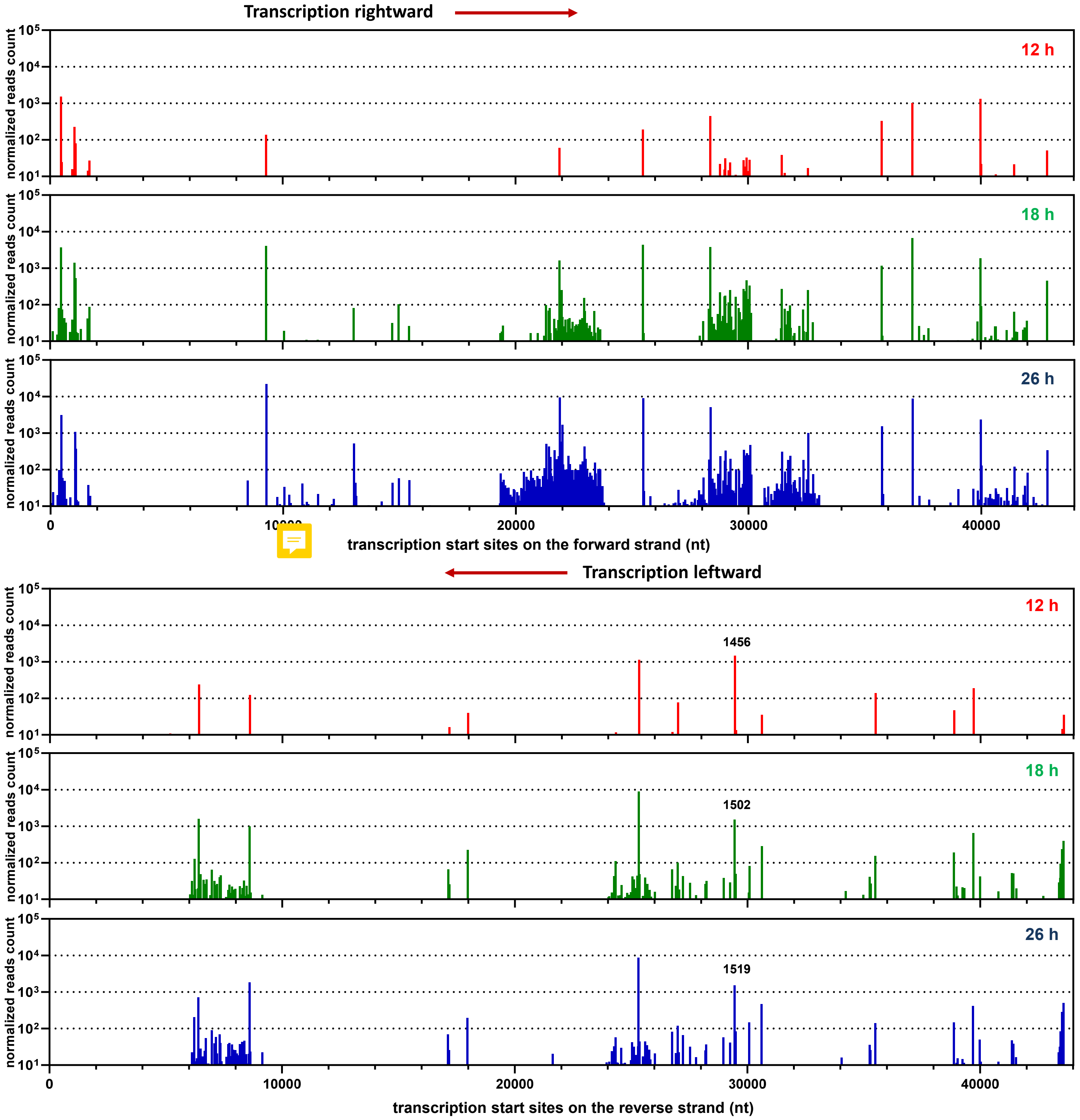

Figure S18. Transcription activity on the forward and the reverse strands of FAdV-4 at 12, 18 or 26 hours post virus infection.
At every time points, the full length reads, which started within a distance of 30-nt to a major transcription start site (TSS), were counted up and treated as the total mRNAs of the TSS. The calculation was carried out by running a previously published perl script as described in the section of Materials and Methods. The total full length reads at that time point (including those from cells and from viruses, Table S1) were divided by 1,000,000 and used to normalize the copy number of viral mRNAs. The answers were the normalized reads count, which represented the promoter activity at a TSS. It could be seen that the normalized reads counts at 29442-nt on the reverse strand were 1456, 1502 and 1519 at 12, 18 and 26 hours post infection, respectively, which were relatively stable.

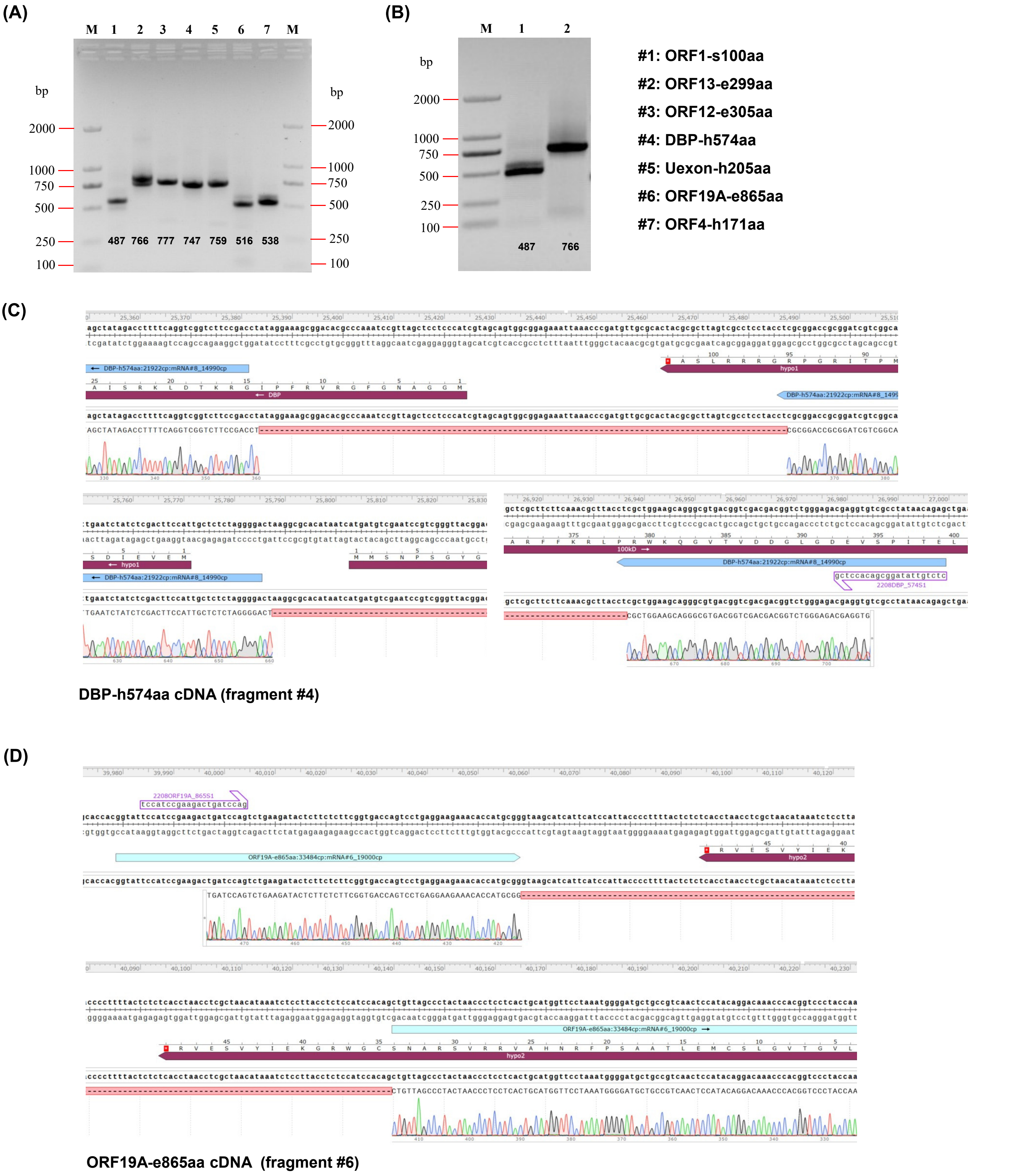

Figure S19. Validation of found introns by RT-PCR and Sanger sequencing**.**
Seven representative ORFs, which spanned introns, were selected. Primer pairs that span introns on the genomic sequence were designed and synthesized. RNA was extracted from chicken LMH cells at 18 hours post infection, treated with DNaseI, and reversely transcribed into cDNA with oligo(dT) as the primer. RT-PCR was performed and the products were resolved in agarose gel after electrophoresis (A). The fragments #1 and #2 were contaminated by intron-containing products. Therefore, target products were excised and recovered from the gel and used as the template for the second round PCR for enriching more PCR product of #1 and #2 (B). All target RT-PCR products were recovered from gel and subjected into Sanger sequencing. The results confirmed the existence of introns and were totally consistent with the data of corrected nanopore full-length cDNA sequencing. The results of Sanger sequencing for DBP-h574aa (C) and ORF19A-e865aa (D) were representatively shown.
